# Co-culture of the bone and bone marrow: a novel way to obtain mesenchymal stem cells with enhanced osteogenic ability for fracture healing in SD rats

**DOI:** 10.1186/s13018-019-1346-z

**Published:** 2019-09-03

**Authors:** Cong Zhu, Mo Sha, Huixiang Jiang, Jianbiao Lin, Weibin Lin, Wenchang Li, Xiaoshan Chen, Guofeng Huang, Zhenqi Ding

**Affiliations:** 1 Center for Orthopedics, Affiliated Southeast Hospital of Xiamen University/909th Hospital of People’s Liberation Army, 269 Zhanghua Middle Road, Zhangzhou, 363000 Fujian Province China; 2 Xiamen University Medical College, Xiang’an South Road, Xiang’an District, Xiamen, 361102 Fujian Province China

**Keywords:** Mesenchymal stem cells, Co-culture, Osteogenic differentiation, Bone fracture, Healing

## Abstract

**Background:**

Mesenchymal stem cells (MSCs) have great potential for the repair and regeneration of bone fracture, but their optimal origins remain controversial.

**Methods:**

Bone marrow-MSCs (BM-MSCs) and bone-bone marrow-MSCs (B-BM-MSCs) were isolated from 12 SD rats, and the morphology, MSC-associated markers, and proliferative capacity of these cells were compared using an inverted microscope, flow cytometry, and CCK-8 assays, respectively. After 14 days of osteoblastic induction, osteoblast phenotypes were detected by ALP and calcium nodule staining, and the expression of BMP-2 and TGF-β1 was observed by western blotting. Then, the rat tibia fracture model was established with 3 groups (*n* = 6 per group), the control, BM-MSC, and B-BM-MSC groups. Computed tomography (CT) imaging was performed to evaluate fracture healing at weeks 2, 4, and 6. Finally, the fractured bones were removed at weeks 4 and 6, and HE staining was performed to evaluate fracture healing.

**Results:**

Although the 2 types of MSCs shared the same cellular morphology and MSC-associated markers, B-BM-MSCs had a higher proliferative rate than BM-MSCs from day 9 to day 12 (*p* < 0.05), and the expression levels of ALP and calcium were obviously higher in B-BM-MSCs than in BM-MSCs after osteogenic induction (*p* < 0.01 and *p* < 0.001, respectively). Western blot results showed that the expression levels of BMP-2 and TGF-β1 in B-BM-MSCs were higher than in BM-MSCs before and after osteogenic induction (*p* < 0.01). In the animal experiments, CT imaging and gross observation showed that B-BM-MSCs had a greater capacity than BM-MSCs to promote fracture healing, as the Lane-Sandhu scores of B-BM-MSCs at weeks 4 and 6 after operation (3.00 ± 0.81 and 9.67 ± 0.94, respectively) were higher than those of BM-MSCs (1.33 ± 0.47 and 6.67 ± 1.25, respectively; both *p* < 0.05). The HE staining results further supported this conclusion.

**Conclusions:**

Taken together, our study results proved that MSCs obtained by co-culturing the bone and bone marrow from SD rats had better proliferative, osteogenic differentiation, and fracture healing capacities than BM-MSCs, perhaps suggesting a novel way to obtain MSCs for bone tissue repair.

## Introduction

Fracture is a common surgical complication that is expensive to treat and has negative effects on individuals and society. In addition, approximately 10% of fractures cannot be cured in a normal way [1, 2]. Orthopedists have adopted many solutions to promote the regeneration of bone tissues, among which stem cell therapy [3–6] plays an important role. Mesenchymal stem cells (MSCs) are a type of adult stem cell that can develop into cells of bone, adipose, cartilage, tendon, ligament, et cetera [7, 8]. MSCs are used as seed cells in tissue engineering transplantation because of their high proliferative capacity, multidirectional differentiation potential, low immunogenicity, and paracrine effects [9]. At present, the available sources of MSCs are the umbilical cords, bone marrow, dental pulp, bone, adipose, et cetera. However, there are currently no excellent methods for obtaining MSCs for fracture treatment have been found yet. For example, it is difficult to apply umbilical cord MSCs in clinical practice due to limited sources [10, 11]. For MSCs derived from the bone marrow (BM-MSCs), the bone marrow has a small number of MSCs and their osteogenic potential is weaker than that of bone MSCs (B-MSCs) [11, 12]. Similarly, adipose-derived MSCs have worse osteogenic potential than B-MSCs [13]. Although B-MSCs can be used as important seed cells for promoting bone regeneration, large amounts of the bone isolated from the body would cause serious secondary damage, severely limiting its clinical application [13, 14]. Other approaches to MSC acquisition also face challenges in sourcing, tumorigenicity control, osteogenic potential, et cetera [15, 16]. Therefore, it is necessary to develop a new approach to extract MSCs with great proliferative capacity and osteogenic potential from various sources while causing minimal damage to the body.

Studies have shown that co-culture of cartilage and MSCs can improve the chondrogenic ability of MSCs [17, 18], and the stimulation of MSCs with fibroblast growth factor can enhance their ability to promote fracture repair of MSCs [19]. These results suggest that MSCs interact with the environment in ways that affect their growth. Meanwhile, the bone and bone marrow co-exist in biological organisms, and MSCs in co-cultures of the bone and bone marrow are more similar to endogenous cells. In this study, we developed a novel way to obtain MSCs by co-culturing the bone and bone marrow (B-BM-MSCs) and explored whether the acquired MSCs are more effective at healing bone tissues.

To address this problem, 2 types of MSCs were isolated from the bone marrow and from co-cultures of the bone and bone marrow, and the cellular characteristics and capacity for fracture healing of the 2 types of cells were compared. Since TGF-β1 and BMP-2 play important regulatory roles in the osteogenic differentiation of mesenchymal stem cells [20, 21], we examined the expression of TGF-β1 and BMP-2 before and after osteogenic induction in both groups and further explored the relevant mechanisms.

## Materials and methods

### Isolation and culture of rat B-BM-MSCs and BM-MSCs

A 6-week-old male SD rat was sacrificed with an injection of 10% chloral hydrate, and the femur and tibia were removed and placed into a sterile Petri dish.

① To obtain B-BM-MSCs, the medullary cavity was washed with PBS mixed with heparin sodium (0.04 mg/ml, H8270, Beijing Solarbio Science & Technology Co., Ltd.) until it appeared clean of all periostea, and the total marrow isolate was collected by centrifugation. Then, the clean femur and tibia were cut into 3 mm × 3 mm bone pellets and placed in a Petri dish with collagenase I (3 mg/mL, c8150, Beijing Solarbio Science & Technology Co., Ltd.). The dish was incubated in a cell incubator (37 °C, 5% CO_2_) for 45 min [19]. At the same time, the bone marrow sample with a cell concentration of 2 × 10^8^/mL to 1 × 10^9^/mL was resuspended in PBS, 5 mL rat mesenchymal cell separation fluid (LGS1072, Tianjin HaoYang HuaKe Biological Technology Co., Ltd.) was added to a 15 mL centrifuge tube, the cell suspension was placed onto the separation liquid surface, and the tube was centrifuged at 450×*g* for 30 min at room temperature. The second layer of the milky white cell layer was placed in another 15 mL centrifuge tube and washed twice with PBS. Then, the cells were filtered through a filter with a pore size of 74 μm, and the filtered liquid was collected into a 6-cm Petri dish containing 6 mL MSC complete medium [DMEM/High Glucose (HyClone, USA) + 10% FBS (No. 04-001-1A, Biological Industries, Israel) + 1% streptomycin/penicillin (100× SV30010, HyClone, USA) + 50 μmol/L β-mercaptoethanol (M8210, Beijing Solarbio Science & Technology Co., Ltd.)]. After a 45-min incubation, 3 pieces of the bone were moved to the same Petri dish, and the dish was placed into a cell incubator. The medium was changed every 48 h, and the cells were maintained in the same medium until they reached approximately 80% confluence. These cells were considered passage 0. Cells were then trypsinized (0.5% trypsin, T8150, Beijing Solarbio Science & Technology Co., Ltd.) and re-cultured for the next passage.

② To obtain BM-MSCs, the cells in the bone marrow were obtained as mentioned above and cultured using the same approach as that for B-BM-MSCs. The isolation and culture of BM-MSCs were guided by the methods reported by Blashki [22].

Two types of P3 MSCs were transfected with lentivirus carrying the green fluorescent protein (GFP) gene (multiplicity of infection = 100). After 12 h, the medium containing the virus solution was discarded, and MSC complete medium was added for 2 or 3 days. The cells were then photographed with a microscope. The sample size was 12.

### Analysis of MSC-associated markers by flow cytometry

The P3 cells were harvested and adjusted to a concentration of 1 × 10^6^/mL prior to staining with antibodies against CD90 (11-0900-81), CD44 (12-0444-80), CD29 (17-0291-80), CD45 (11-0461-80), CD31 (25-0310-80) (Thermo Fisher Scientific, Ebioscience, USA), and CD106 (lot 130-103-684, Miltenyi Biotec, Germany). Fluorescence-activated cell identification was performed with flow cytometry (Beckman USA), and the data were analyzed with CytExpert (Tree Star, Ashland, OR, USA). The sample size was 12.

### Proliferation ability of MSCs from different sources

After a 9-day cultivation, the concentration of the 2 types of MSCs was adjusted to 2 × 10^4^ cells/mL, and these cell solutions inoculated in a 96-well cell culture plate with 100 μL/well. After culture for 1, 2, 3, and 4 days, 10 μL CCK-8 reagent (FC101-03, TransGen Biotech, Beijing, China) was added to each well, and the plates were incubated at 37 °C with 5% CO_2_ for 2 h. The OD values were determined at 450 nm with a microplate reader (Bio-Rad, USA). The cell proliferation rate was calculated as follows: rate (day *X*) = [OD450 (day *X*)-OD450 (day *X*-1)]/OD450 (day *X*-1). The sample size was 6.

### Osteogenic differentiation ability of MSCs from different sources

The P3 MSCs from the 2 different sources were seeded in 6-cm dishes with 2 × 10^5^ cells per dish. The cells were cultured at 37 °C with 5% CO_2_ for 24 h. Then, the original medium was replaced with osteogenic induction medium containing 10^−2^ mol/L sodium glycerophosphate (MB3195, Dalian Meilun Biotech Co., Ltd.), 10^−7^ mol/L dexamethasone (MB1434, Dalian Meilun Biotech Co., Ltd.), and 3 × 10^−4^ mol/L vitamin C (MB3195, Dalian Meilun Biotech Co., Ltd.). The induction medium was changed every 2 days, and the cells were induced for 14 days. Then, the cells were fixed, ALP and calcium nodules were stained with the modified Gomori Calcium-Cobalt method (DE0001, Beijing Leagene Biotech Co., Ltd.), and alizarin red staining (DS0002, Beijing Leagene Biotech Co., Ltd.) was performed according to the manufacturer’s instructions. The ratio of the positive area under each high-power field (RPA-HPT) was used to evaluate ALP expression and calcium nodules. The sample size was 6.

### The expression of TGF-β1 and BMP-2 in the 2 types of MSCs before and after osteogenic induction

Cell samples were isolated with RIPA buffer (RIPA:PMSF = 100:1, R0020 and P8340, Beijing Solarbio Science & Technology Co., Ltd), and the total protein content was measured with the bicinchoninic acid protein assay kit (Thermo Scientific, Waltham, MA, USA). After the addition of loading buffer, the samples were boiled for 5 min for protein denaturation. Then, the samples were separated by sodium dodecyl sulfate-polyacrylamide gel electrophoresis (SDS-PAGE) in a 12% gel under a constant voltage of 80 V for 30 min followed by a constant voltage of 110 V until the samples reached the bottom of the separation gel. Proteins were resolved by denaturing SDS-PAGE followed by transfer onto a nitrocellulose membrane. GAPDH (AB0037, Shanghai Abways Biotechnology Co., Ltd.) was used as the loading control. The membranes were incubated overnight at 4 °C with primary antibodies against TGF-β1 (1:1000, arg10002, Arigo Biolaboratories, Taiwan, China), BMP-2 (1:500, arg65980, Arigo Biolaboratories, Taiwan, China), and GAPDH (1:5000). The membranes were incubated with secondary antibodies (HS101-01 and HS201-01, Beijing TransGen Biotech Co., Ltd.) conjugated with horseradish peroxidase for 1 h. Target proteins were detected by an enhanced chemiluminescence system (4AWO12-050, Beijing 4A Biotech, Co., Ltd.) prior to development on X-ray film and photographic imaging to visualize the results. The sample size was 4.

### Rat fracture model

**Fig. 1:**
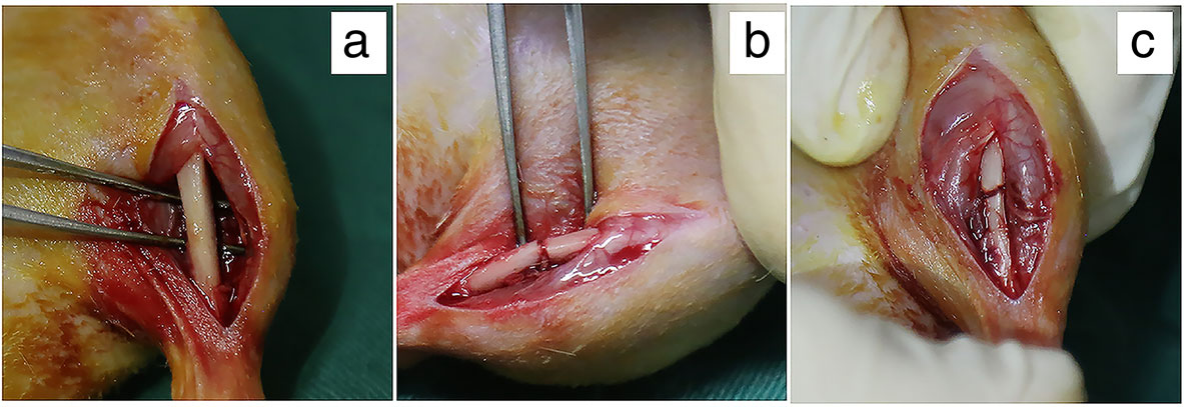
Rat bone fracture model construction. **a** The tibia was dissected with blunt tweezers. **b** The tibia was cut in the middle. **c** The fracture was well aligned

Male SD rats weighing approximately 180 g to 220 g were anesthetized with 10% chloral hydrate. The right lower limbs were depilated with hair removal cream and disinfected with 2% iodophor and 75% alcohol. A longitudinal incision was made from the medial part 3 mm below the tibial platform to the medial malleolus at 10 mm, and the surface fascia at the incision site was cut. The tibia was dissected (Fig. 1a) and then cut in the middle with a wire clamp (Fig. 1b). The process required care to avoid damaging the fibula. The fracture area was washed with iodine and confirmed to be fully aligned (Fig. 1c). Then, 10 μL PBS or 3 × 10^6^ cells (dissolved in 10 μL PBS) were injected into the fracture sites of the control, BM-MSC, and B-BM-MSC groups with a 1-mL syringe. Then, the surface fascia and skin were sutured, the incision was wrapped with sterile gauze and fixed with plaster, and the treatment outcome was evaluated at subsequent time points. The sample size was 6.

### Observation of the fracture healing process

At 2, 4, and 6 weeks after the operation, the fracture healing process was observed by computed tomography (CT) imaging, whereas visual inspection and hematoxylin and eosin (H&E) staining were used to evaluate the fracture healing process at weeks 4 and 6 after the operation. The sample size was 6.

### Statistical analysis

The results are expressed as the mean ± standard deviation. Prism 5.0 (GraphPad Software Inc., San Diego, CA, USA) was used for statistical analysis. Statistical comparisons among different groups were performed with one-way or two-way analysis of variance (ANOVA). *p* < 0.05 indicated a statistical significance.

## Results

### The morphological features of B-BM-MSCs and BM-MSCs

**Fig. 2:**
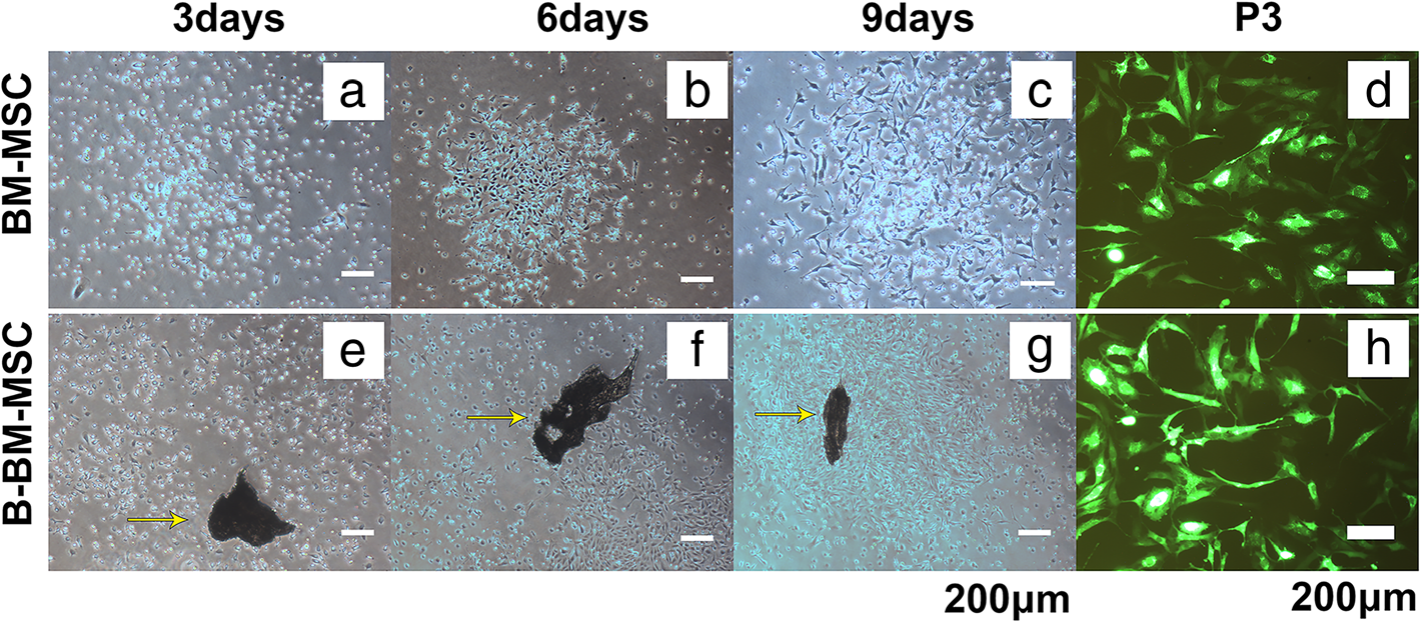
The morphological features of MSCs from different sources were observed by an inverted microscope. The early-stage B-BM-MSCs (**e**, **f**) were spindle-shaped, triangle-shaped, and polygon-shaped, while the early-stage BM-MSCs (**a**, **b**) were polygon-shaped and round. However, the 2 types of P3 MSCs expressing GFP shared the same morphology (**d**, **h**). *n* = 12. Yellow arrows indicate the bone. Scale bar: 200 μm. P, passage

The early-stage BM-MSCs (Fig. 2a, b) were polygon-shaped and round, while the early-stage B-BM-MSCs (Fig. 2e, f) were spindle-shaped, triangle-shaped, and polygon-shaped; some cells were clustered, and others were round and scattered. Both BM-MSCs and B-BM-MSCs tended to become fusiform or streamlined (Fig. 2c, g) over time. The primary BM-MSCs reached 80 to 90% confluence at days 14 to 16, while the primary B-BM-MSCs reached 80–90% confluence at days 9 to 11. However, mature BM-MSCs and B-BM-MSCs shared similar morphology (Fig. 2d, h) after GFP lentiviral transfection, and both adopted a fusiform or streamlined shape.

### MSC-associated marker expression in B-BM-MSCs and BM-MSCs

**Fig. 3:**
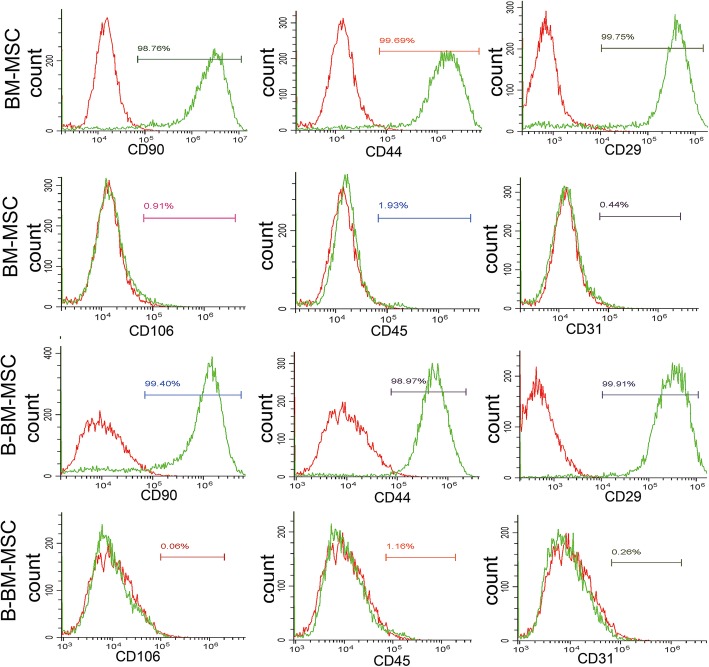
Immunophenotyping of MSCs from different sources by flow cytometry assays. Two types of P3 MSCs were chosen for immunophenotyping. According to the flow cytometry results, CD90 (99.18% ± 0.15%), CD44 (98.47% ± 0.89%), and CD29 (99.39% ± 0.36%) were prominently expressed (> 97%), whereas CD106 (0.11% ± 0.03%), CD45 (1.58% ± 0.31%), and CD31 (0.23% ± 0.02%) were barely expressed (< 2%) in B-BM-MSCs, which was consistent with the results of BM-MSCs. Stained cells are represented in green, whereas unstained cells are in red. *n* = 12.

According to the flow cytometry results, CD90 (99.18% ± 0.15%), CD44 (98.47% ± 0.89%), and CD29 (99.39% ± 0.36%) were prominently expressed (> 97%) in B-BM-MSCs, whereas CD106 (0.11% ± 0.03%), CD45 (1.58% ± 0.31%), and CD31 (0.23% ± 0.02%) were barely expressed (< 2%) in B-BM-MSCs. These results were consistent with the results of BM-MSCs. These results indicated that B-BM-MSCs were successfully separated and cultured. It could be concluded that all the obtained MSCs were of high purity since MSC-associated markers were highly prevalent among these cells in every situation (Fig. 3).

### Proliferation ability of B-BM-MSCs and BM-MSCs in vitro

**Fig. 4:**
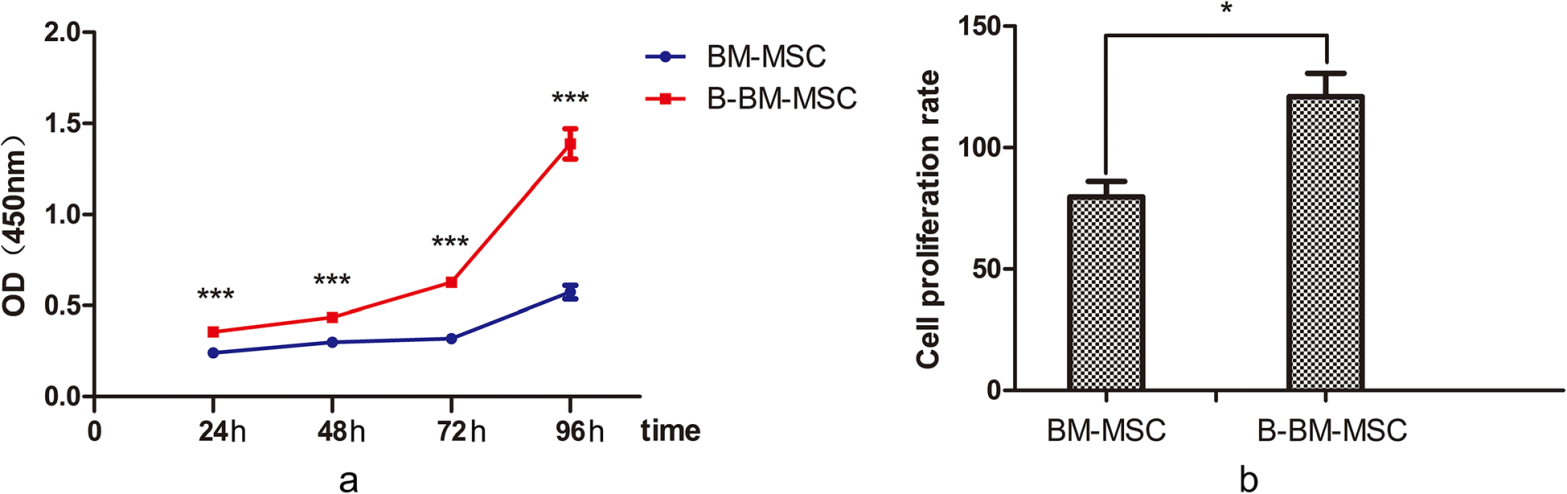
The proliferation of MSCs from different sources was detected by CCK-8 assays. The proliferative capacity of B-BM-MSCs was greater than that of BM-MSCs, as the cell proliferation rates on day 4 were 110.94% ± 17.02% and 79.95% ± 11.21%, respectively, (*p* < 0.05). *n* = 6. *p < 0.05, **p < 0.01, ***p < 0.001

From 24 to 96 h, the proliferative capacity of B-BM-MSCs was higher than that of BM-MSCs (Fig. 4a); the corresponding cell proliferation rates on day 4 were 110.94% ± 17.02% and 79.95% ± 11.21% (*p* < 0.05). In addition, the proliferative capacity of B-BM-MSCs was greater than that of BM-MSCs (Fig. 4b). These cells all showed the greatest proliferation ability from 72 to 96 h.

### Osteogenic differentiation ability of B-BM-MSCs and BM-MSCs in vitro

**Fig. 5:**
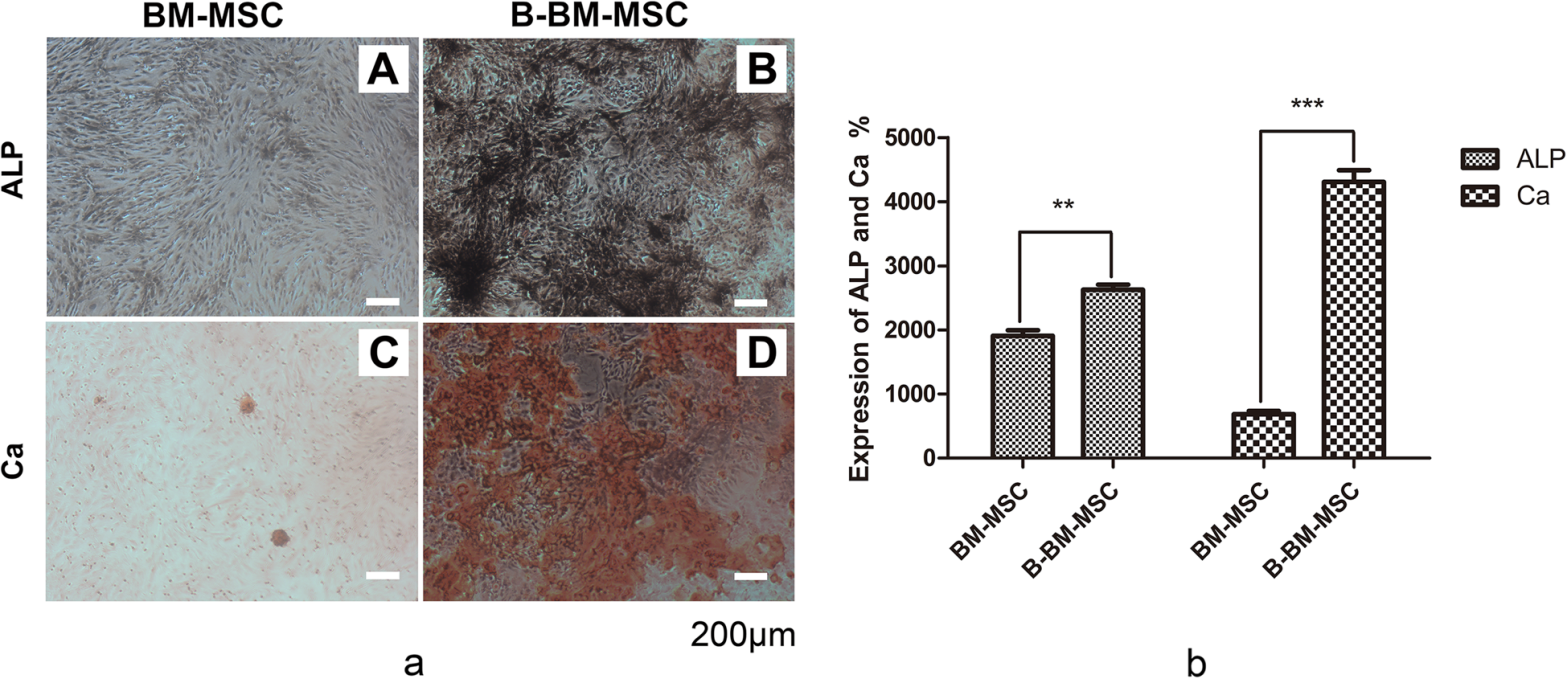
**a** (A-D): The expression levels of ALP and calcium in MSCs from different sources were detected by the modified Gomori Calcium-Cobalt method and alizarin red staining. **b**: Statistical analysis results. The expression levels of ALP and calcium were obviously higher in B-BM-MSCs than in BM-MSCs (ALP 26.27% ± 1.11% vs 19.08% ± 1.23%, *p* < 0.05; calcium 43.05% ± 2.63% vs 6.81% ± 0.72%, *p* < 0.001; ratio of positive area under each high-power field). *n* = 6. Magnification: × 60. Scale bar: 200 μm. **p < 0.01, ***p < 0.001

The results of alizarin red staining and the modified Gomori Calcium-Cobalt method showed considerable ALP expression and numerous calcium nodules after osteogenic induction for 14 days (Fig. 5a). The results showed that B-BM-MSCs (RPA-HPT, 26.28% ± 1.11%) induced more ALP-stained black plaques than BM-MSCs (RPA-HPT, 19.08% ± 1.23%) (*p* < 0.05), and B-BM-MSCs generated more calcium nodules (RPA-HPT, 43.05% ± 2.62%) than BM-MSCs (RPA-HPT, 6.81% ± 0.72%) (*p* < 0.001) (Fig. 5b). These findings showed that the osteogenic differentiation ability of B-BM-MSCs was better than that of BM-MSCs in vitro.

### Expression of BMP-2 and TGF-β1 in B-BM-MSCs and BM-MSCs before and after osteogenic induction

**Fig. 6:**
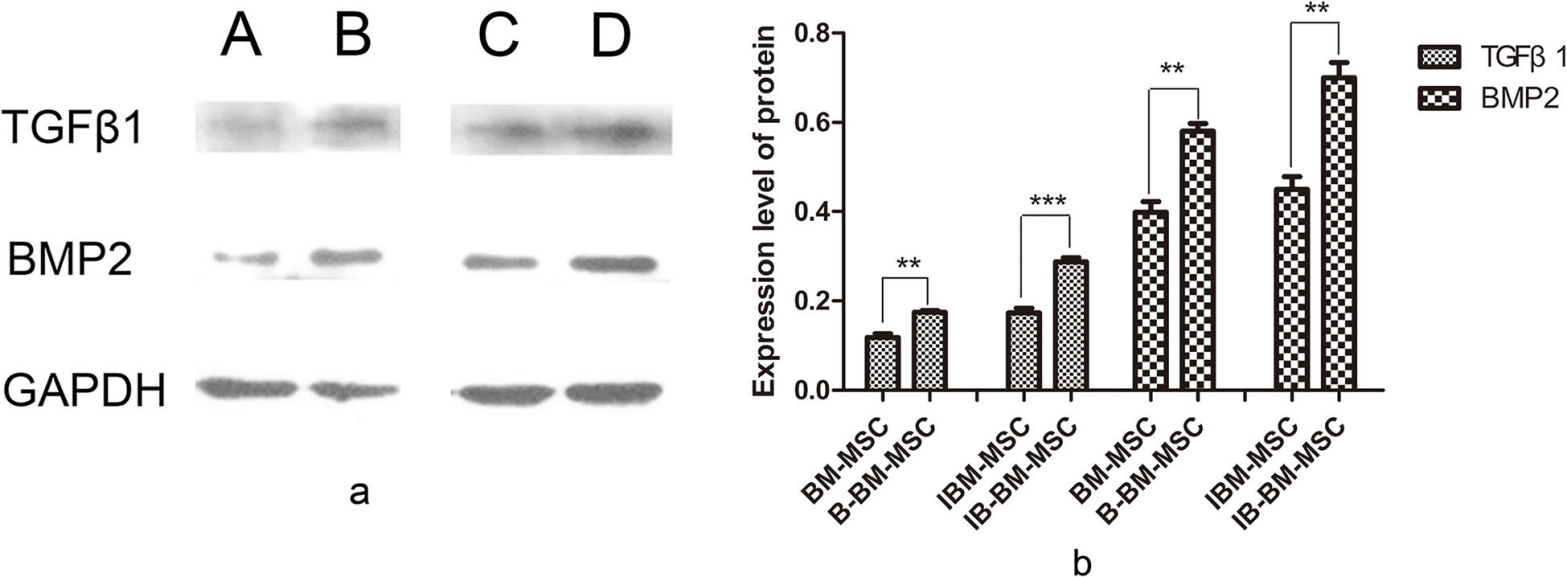
**a**, **b** BMP-2 and TGF-β1 expression in MSCs from different sources before and after osteogenic induction was detected by western blot. The expression of both BMP-2 and TGF-β1 was higher in B-BM-MSCs than in BM-MSCs before and after 14 days of osteogenic induction (*p* < 0.05 and *p* < 0.001, respectively). *n* = 4. *p < 0.05, **p < 0.01, ***p < 0.001. A and B show the expression levels of BM-MSCs and B-BM-MSCs, respectively, before osteogenic induction, and C and D show the expression of the indicated molecules in BM-MSCs and B-BM-MSCs, respectively, after osteogenic induction. IBM-MSC, osteogenic-induced bone marrow mesenchymal stem cell; IB-BM-MSC, osteogenic induced-bone-bone marrow mesenchymal stem cell

Western blot results showed that the expression of both TGF-β1 and BMP-2 was higher in B-BM-MSCs than in BM-MSCs before osteogenic induction. After a 2-week osteogenic induction, the expression of TGF-β1 and BMP-2 remained higher in B-BM-MSCs compared to BM-MSCs (Fig. 6). This finding further confirmed that the osteogenic potential of B-BM-MSCs was greater than that of BM-MSCs in vitro.

### CT imaging observation of bone fracture healing

**Fig. 7:**
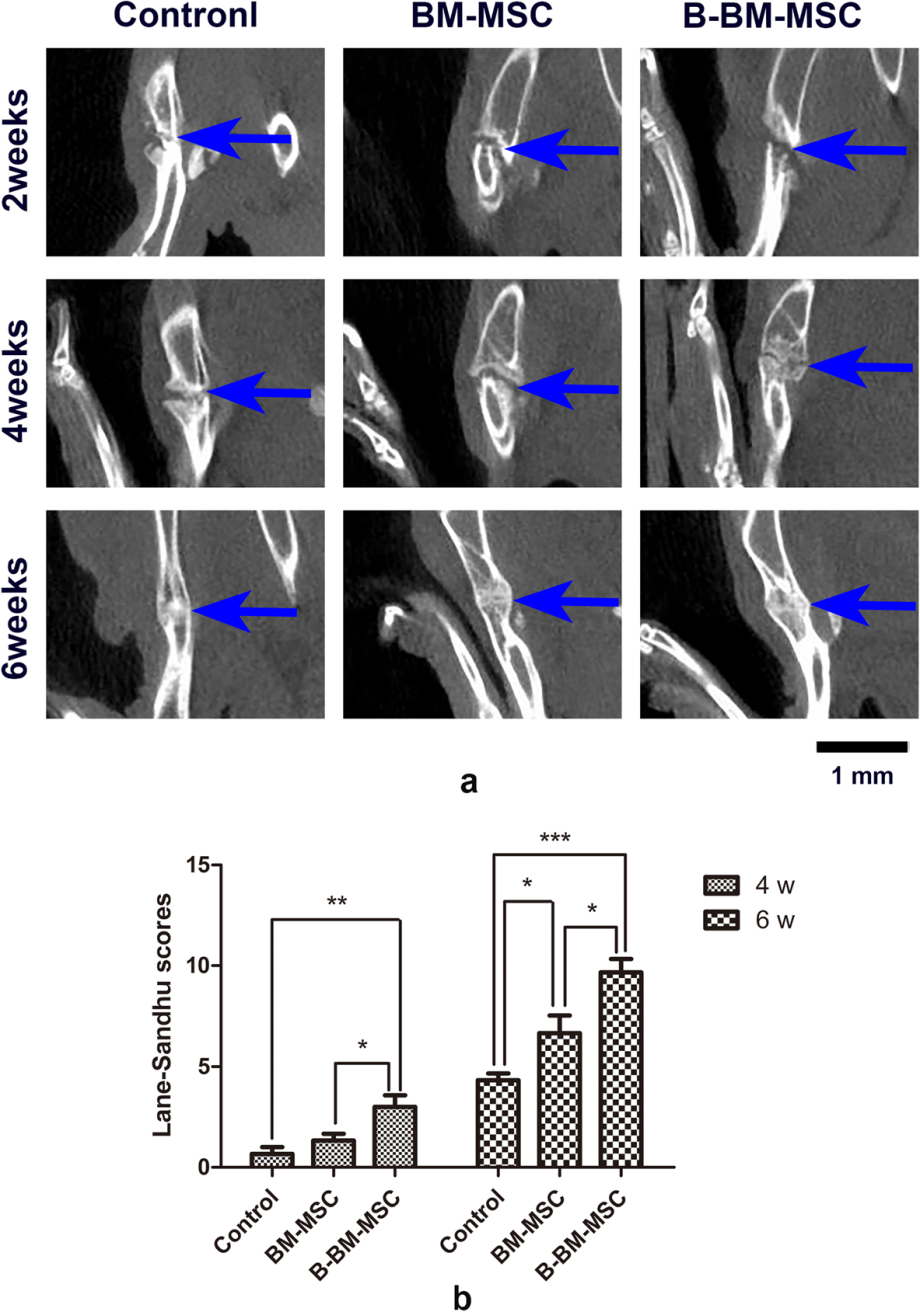
**a**, **b** Healing in the rat fracture model by MSCs from different sources was detected by CT imaging. CT imaging showed that B-BM-MSCs had a stronger ability than BM-MSCs to promote fracture healing in vivo. Lane-Sandhu score analysis after the 4th and 6th weeks showed that B-BM-MSCs scored higher than BM-MSCs (*p* < 0.05). *n* = 6. *p < 0.05, **p < 0.01, ***p < 0.001

In the 2nd week after the operation, the control, BM-MSC, and B-BM-MSC groups all showed little callus formation at the bone fracture sites, while the number of calluses increased in week 4 after the operation in all 3 groups. In the 6th week after the operation, there was substantial new bone formation in the B-BM-MSC group, and the medullary cavity was recanalized, indicating good regeneration, while remodeling was not prominent in the control group or the BM-MSC group (Fig. 7a). Lane-Sandhu score analysis (Fig. 7b) after the 4th and 6th weeks showed that B-BM-MSCs (3.00 ± 0.81 and 9.67 ± 0.94, respectively) scored higher than BM-MSCs (1.33 ± 0.47 and 6.67 ± 1.25, respectively) (*p* < 0.05). This finding indicated that B-BM-MSCs had a greater ability than BM-MSCs to promote fracture healing in vivo.

### Gross observation of bone fracture repair

**Fig. 8:**
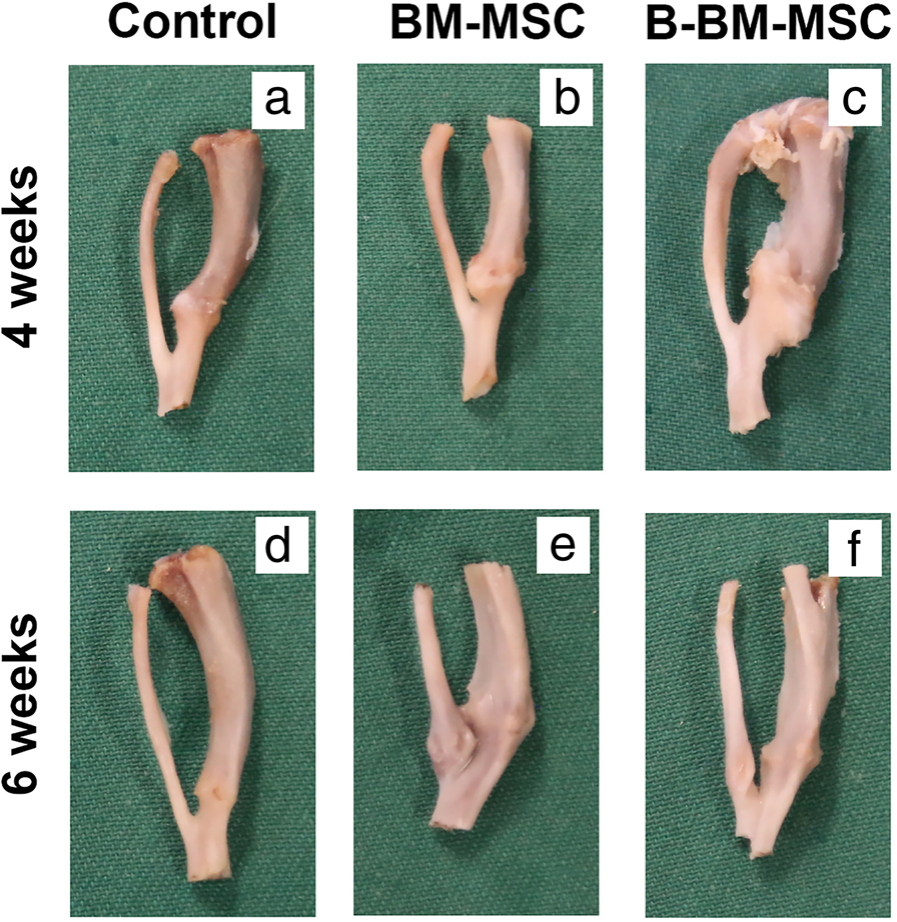
**a**–**f**: Healing in the rat fracture model by MSCs from different 2 sources was detected by gross observation. Bone specimens were collected 4 and 6 weeks after construction of the rat fracture model. *n* = 6

To confirm our CT imaging findings, we collected bone specimens 4 and 6 weeks after construction of the rat fracture model. In the control group, some new calluses formed around the fracture area 4 weeks after surgery (Fig. 8a). The fracture site showed on a large number of soft tissue connections, and the healing condition was not good enough. At the 6th week, the peripheral osteophytes had basically been absorbed, and the cortical bones were connected (Fig. 8d). The situation in the BM-MSC group (Fig. 8b, e) was slightly better than that in the control group, while the fracture healing in the B-BM-MSC group (Fig. 8c, f) at weeks 4 and 6 was significantly better than that in the control group. The sample size of this experiment was 6. Representative data from one animal per group are shown, and similar results were obtained for the other animals in each group. At the 4th and 6th weeks, fracture healing in the B-BM-MSC group was better than that in the BM-MSC group, while fracture healing in the BM-MSC group was better than that in the control group. These data indicated that both BM-MSCs and B-BM-MSCs can effectively promote fracture healing, although B-BM-MSCs produced superior results to BM-MSCs.

### Histological assessment of bone regeneration

**Fig. 9:**
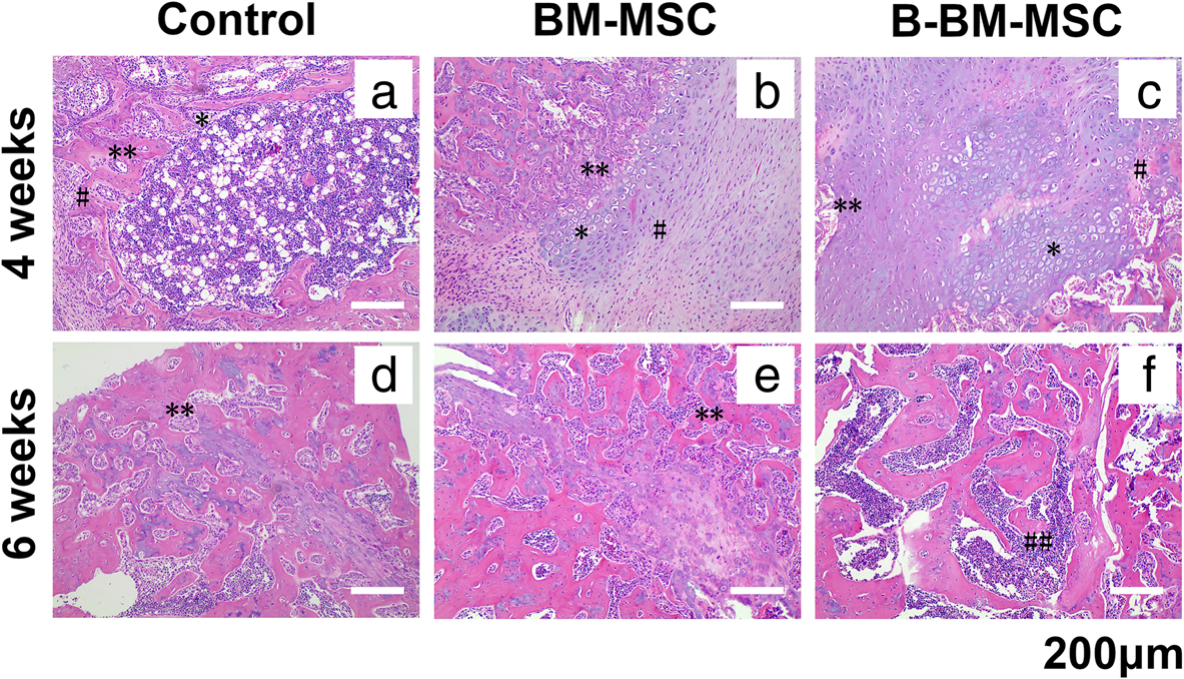
**a**–**f**: Healing in the rat fracture model by MSCs from different sources was detected by H&E staining. H&E staining of bone specimens collected 4 and 6 weeks after construction of the rat fracture model. B-BM-MSCs had a stronger ability than BM-MSCs to promote fracture healing. *n* = 6. *chondrocytes, #osteogenic cells, **osteoblasts, ##hematopoietic cells

At the 4th week, large numbers of chondrocytes and osteogenic cells were observed in trabeculae, large populations of osteoblasts were observed around trabeculae, and hematopoietic cell proliferation in trabeculae was extremely active in the B-BM-MSC and BM-MSC groups (Fig. 9b, c). In the control group (Fig. 9a), some osteoblasts were also observed around trabeculae, whereas hyperplasia of chondrocytes and osteogenic cells was not obvious. At the 6th week, in the control (Fig. 9d) and BM-MSC groups (Fig. 9e), large numbers of osteoblasts were visible, and cells in trabeculae were tightly packed, whereas in the B-BM-MSC group (Fig. 9f), trabeculae were mainly filled with hematopoietic cells, cells in the medullary cavity were loosely packed, and the trabeculae were more mature. The sample size of this experiment was 6, and consistent results were obtained in other groups. At the 4th and 6th weeks, fracture healing and reconstruction were better in the B-BM-MSC group than in the BM-MSC group, which showed better results than the control group in these 2 aspects. This finding further proved that B-BM-MSCs had a greater ability than BM-MSCs to promote fracture healing.

## Discussion

In this study, we obtained MSCs through the co-culture of bone and bone marrow for the first time and found that B-BM-MSCs have better proliferative, osteogenic differentiation, and fracture healing capacities than BM-MSCs. We suggest that co-culturing the bone and bone marrow might be a useful method for obtaining seed cells for bone tissue repair.

Fernandez-Moure compared MSCs from the human bone and from the human bone marrow and concluded that CBF-MSCs had a weaker proliferative ability than BM-MSCs, but BM-MSCs had a significantly better osteogenic differentiation ability [13]. In terms of cell proliferation, we reached different conclusions than Fernandez-Moure. The reasons might be that MSCs were traditionally obtained through density gradient centrifugation, but some cancellous bone or soft tissue may be retained in this process [22]. For the isolation in this study, we used a filter with a diameter of 74 μm to exclude cancellous bone and soft tissue. Alternatively, the discrepant results might be caused by different species used in our experiments. Daniel Blashki compared the proliferative ability of B-MSCs and BM-MSCs from rats [22], and our results are consistent with his conclusions. Concerning osteogenic differentiation, our results are similar to those reported by Fernandez-Moure in certain aspects.

Corradetti et al. demonstrated that the environment has an important effect on the differentiation direction of B-MSCs and BM-MSCs [14], and some research has shown that the environment also somewhat determines the differentiation trend of MSCs [14, 23–25]. We suggest that the change in the environment of MSCs co-cultured with the bone and bone marrow resulted in improved proliferative and osteogenic differentiation abilities. TGF-β1 promotes the differentiation of precursor osteoblasts in the early stage [20, 26], and BMP-2 is important in osteogenic differentiation and indispensable for the osteogenic differentiation of MSCs [21, 27]. The results of this study showed that B-BM-MSCs had higher TGF-β1 and BMP-2 expression than BM-MSCs. We concluded that the co-culture of the bone and bone marrow might enhance the osteogenic potential by increasing the expression of TGF-β1 and BMP-2 in MSCs. Wang et al. found that the upregulation of TGF-β promoted tendon-to-bone healing after anterior cruciate ligament reconstruction with BM-MSCs [28], which further supports our conclusion.

The acquisition of B-MSCs can cause secondary damage to patients in clinical applications [13], while BM-MSCs can be easily obtained through bone marrow biopsy. Therefore, obtaining B-BM-MSCs through co-culture of a small amount of the bone with a relatively large amount of the bone marrow may avoid serious secondary damage to patients and ensure that the obtained MSCs have good proliferative activity and osteogenic potential.

Although the benefits of transplanting MSCs to promote fracture healing have been confirmed, there are still some problems remaining to be solved in clinical applications. Vadala et al. found that B-MSC injection in degenerated intervertebral disks in rats might induce osteophyte formation [29]. Thus, it is important to explore ways of inducing the localized differentiation of transplanted cells. In this study, we found that co-culture of tissues from different sources could induce the directional differentiation of MSCs to some extent, but this did not completely solve this problem. In addition, we found that using 3 pieces of 3 mm × 3 mm bone in 6-cm Petri dishes was helpful, but it is still unclear whether there is a better proportion of the bone in the culture system. We found that TGF-1 and BMP-2 in the co-culture of the bone and bone marrow played regulatory roles in promoting the proliferation, osteogenic differentiation, and fracture healing of MSCs, but the specific mechanism still needs further research. Therefore, further investigations are required to develop more satisfactory ways to use MSC transplantation to promote fracture healing.

## Conclusion

In this study, we propose a novel way to obtain MSCs by co-culturing the bone and bone marrow from SD rats; these MSCs shared the same morphologic features and MSC-associated markers as traditional BM-MSCs, while their proliferative capacity and osteogenic potential were higher, and they successfully promoted fracture healing after injection into the fracture site. Therefore, this method may provide a promising source of MSCs for bone tissue engineering and clinical fracture treatment.

## Data Availability

The datasets used and/or analyzed during the current study are available from the corresponding author on reasonable request.

## Abbreviations

ALP: Alkaline phosphatase
B-BM-MSCs: Bone-bone marrow mesenchymal stem cells
bFGF: Basic fibroblast growth factor
BM-MSCs: Bone marrow mesenchymal stem cells
BMP-2: Bone morphogenetic protein-2
B-MSCs: Bone mesenchymal stem cells
CBF-MSCs: Mesenchymal stem cells from cortical bone fragments
CCK-8: Cell counting kit-8
CT: Computed tomography
DMEM: Dulbecco’s modified Eagle’s medium
FBS: Fetal bovine serum
GAPDH: Glyceraldehyde-3-phosphate dehydrogenase
GFP: Green fluorescent protein
HE: Hematoxylin and eosin
MSCs: Mesenchymal stem cells
OD: Optical density
P: Passage
PBS: Phosphate-buffered saline
PMSF: Phenylmethanesulfonyl fluoride
RIPA: Radioimmunoprecipitation assay
RPA-HPT: Ratio of positive area under each high-power field
Runx2: Runt-related transcription factor 2
SD: Sprague Dawley
SDS-PAGE: Sodium dodecyl sulfate-polyacrylamide gel electrophoresis
Smads: Mothers against decapentaplegic homologs
TGF-β1: Transforming growth factor beta 1
